# Effectiveness of Single Fetal Membrane Sweeping in Reducing Elective Labor Induction for Postdate Pregnancies (38+0 to 40+6 Weeks): A Randomized Controlled Trial

**DOI:** 10.7759/cureus.58030

**Published:** 2024-04-11

**Authors:** Eustace Ehikioya, Onyinyechukwu B Nwachukwu, Okelue E Okobi

**Affiliations:** 1 Obstetrics and Gynaecology, Edo Specialist Hospital, Benin, NGA; 2 Neurosciences and Psychology, California Institute of Behavioral Neurosciences & Psychology, Fairfield, USA; 3 Family Medicine, American International School of Medicine, Georgetown, GUY; 4 Family Medicine, Larkin Community Hospital Palm Springs Campus, Miami, USA; 5 Family Medicine, Medficient Health Systems, Laurel, Maryland, USA; 6 Family Medicine, Lakeside Medical center, Belle Glade, USA

**Keywords:** perinatal outcomes, maternal outcomes, gestation, membrane sweeping, outcome postdate pregnancy, incidence, elective labor induction, postdate pregnancy, post term pregnancy

## Abstract

Background: Postdate pregnancy is characterized by a heightened risk for both maternal and perinatal complications. Owing to the risks, clinicians frequently turn to elective labor induction as a management strategy for postdate pregnancies. However, patients are increasingly informed and apprehensive about this approach and its associated risks. This has prompted a search for alternative management methods that may encourage spontaneous labor in pregnant women. One such approach is the use of fetal membrane sweeping, a method known to increase the likelihood of spontaneous labor onset. Yet, it remains unclear whether a single fetal membrane sweeping procedure can effectively reduce the need for elective labor induction in postdate pregnancies while minimizing risks to both the mother and fetus.

Objectives: The primary objective of this study was to assess the efficacy of a single fetal membrane sweeping procedure conducted between 38+0 and 40+6 weeks of gestation in reducing the rate of elective labor induction among postdate pregnancies at Central Hospital Benin City, Nigeria. Secondary objectives included evaluating the impact of membrane sweeping on maternal and perinatal outcomes.

Methodology: This open-label superiority randomized controlled study was carried out from June 2020 to March 2021, following ethical approval from the Hospital Management Board (HMB). One hundred and forty eligible participants, without contraindications to vaginal delivery, were randomly assigned to one of two groups. The first group received a single fetal membrane sweeping procedure between 38+0 and 40+6 weeks of gestation, while the control group underwent vaginal examination only to assess the Bishop score. Participants were monitored until delivery. Data analysis was performed. Results were considered statistically significant at p < 0.05.

Results: The implementation of a single fetal membrane sweeping procedure effectively reduced the incidence of elective labor induction. Specifically, the membrane sweep group exhibited a significantly lower rate of elective labor induction compared to the control group (9.0% vs. 27.1%; p=0.0083). Moreover, a substantial proportion of the treatment group (91.4%) experienced spontaneous labor, while the control group reported a rate of 72.9%. The difference was statistically significant (p=0.0054). Notably, the control group exhibited a significantly longer mean time interval from recruitment to delivery (10.67±3.51 days) than the membrane sweeping group (3.64±4.123 days; p<0.05). Also, postdate women in the membrane sweep group were less likely to require cervical ripening with Foley's catheter than those in the control group (33.3% vs. 100%; RR: 0.33 (0.11-1.03); p=0.0057). Still, maternal satisfaction was significantly higher in the membrane-sweeping group (p<0.01). No significant differences were noted across the groups in maternal and neonatal outcomes.

Conclusion: In low-risk term pregnancies, a single fetal membrane sweeping procedure is a superior alternative to no membrane sweeping in reducing the rate of elective labor induction for postdate pregnancies and in shortening the duration of term pregnancy.

## Introduction

Membrane sweeping is considered a less invasive mechanical method for initiating labor [1,2]. It involves the digital separation of the forewater membrane from the lower uterine segment, a simple and cost-effective procedure that plays a pivotal role in transitioning from pregnancy maintenance to spontaneous labor onset. This procedure has the potential to reduce the occurrence of postdate pregnancies and the necessity for elective labor induction [3-6]. Presently, the prevalence of labor induction is steadily increasing in Nigeria, particularly for postdate pregnancies [7-8]. In developed countries, up to one in four deliveries occurs following labor induction [9-10]. In Nigeria, reported rates were 6.6% in Maiduguri, in Borno State, North-eastern Nigeria, in 2008 [5] and 11.5% in Enugu state, South-eastern Nigeria [9], with postdate pregnancy being a common indication for labor induction [11]. This practice has been shown to lower the risk of stillbirth and early neonatal death [11-13].

Postdate pregnancy refers to pregnancies that extend beyond 40 weeks gestation [14, 15], posing potential risks to the fetus due to reduced placental function, which can result in fetal asphyxia and demise [15,16]. The American College of Obstetricians and Gynecologists has categorized term pregnancies into late-term (41+0 to 41+6 weeks) and post-date (42+0 weeks or beyond) pregnancies [8,17]. Thus, the incidence of postdate pregnancies varies across regions, possibly due to differences in diagnostic and management approaches. The use of early ultrasound for accurate pregnancy dating is believed to decrease the occurrence of postdate pregnancies compared to relying solely on the last menstrual period [18-19]. Postdate pregnancies are associated with risks such as meconium aspiration syndrome, fetal acidemia, and neonatal morbidity, including birth injuries, asphyxia, hypoglycemia, encephalopathy, and perinatal death [20-26].

Studies have indicated that perinatal mortality risk sharply rises after 40 weeks gestation [27], with stillbirth risks estimated at one in 926 at 40 weeks, one in 826 at 41 weeks, one in 769 at 42 weeks, and one in 633 at 43 weeks gestation [28]. Maternal risks linked to postdate pregnancies include increased rates of obstructed labor (2%-7% at 40 weeks to 9%-12% beyond 42 weeks), third and fourth-degree perineal lacerations due to fetal macrosomia (increasing from 2.6% at 40 weeks to 3.3% at 41 weeks), and higher rates of operative vaginal delivery and cesarean sections (from 7% at 40 weeks to 14% after 42 weeks) [29-32]. The emotional toll of postdate pregnancies, including anxiety, frustration, and marital discord, is often underestimated [32-34]. Preventing postdate pregnancies is crucial to avoiding these complications.

Additionally, the management of postdate pregnancies presents challenges to healthcare providers, patients, and families. The Royal College of Obstetricians and Gynecologists, London, United Kingdom, and the National Institute for Health and Care Excellence, London, United Kingdom, recommend labor induction between 41 and 42 weeks gestation to prevent complications associated with postdate pregnancies [25]. Inducing labor before 42 weeks gestation has the potential to prevent these complications, but it can impact the birthing experience for women [33]. The procedure often occurs when labor wards are busiest, increasing the strain on staff. It involves several steps, including cervical ripening for unfavorable cervix and prolonged ward admission, which can cause stress and anxiety for women.

While opinions differ on the best cervical ripening methods, the degree of cervical ripening is closely tied to successful vaginal delivery [35]. Non-pharmacologic approaches to cervical ripening and labor induction have included various methods such as herbal compounds, castor oil, hot baths, enemas, sexual intercourse, breast stimulation, acupuncture, acupressure, transcutaneous nerve stimulation, and mechanical and surgical modalities [36]. Among these non-pharmacologic methods, only mechanical methods like stripping of the fetal membranes and amniotomy have demonstrated efficacy for cervical ripening [37].

Membrane sweeping is believed to increase phospholipase A2 and prostaglandin F2a activity and exert mechanical stretch on the cervix, thereby initiating cervical ripening [38]. This procedure involves gently sweeping or stripping the fetal membranes by inserting the index finger of the right hand through the internal cervical os and moving it in a circular motion (360 degrees) to detach the inferior pole of the membranes from the lower uterine segment [38]. Thus, being a mechanical process, membrane sweeping entails the clinician inserting one or two fingers into the patient’s cervix and making use of continuous circular sweeping movement with the objective of detaching the membrane poles from lower uterine segments. The process leads to the release of endogenous prostaglandins and hormones that promote effacement and dilation, which, in turn, leads to cervical ripening and uterine contraction initiation. Following membrane sweep, women are usually sent home to await the spontaneous onset of labor, and a good number of them return in the spontaneous onset of labor, thereby avoiding formal labor induction [38]. Furthermore, speculatively, most deliveries in sub-Saharan Africa are conducted by traditional birth attendants (TBAs), who are not adequately trained to carry out induction. This study shows that membrane sweeping reduces the incidence of induction of labor and its consequences

Membrane sweeping is a non-invasive procedure suitable for situations where immediate induction is not required. It has the potential to hasten labor onset and reduce the need for elective labor induction in postdate pregnancies. Reported adverse effects of membrane sweeping include mild vaginal bleeding, discomfort, Braxton Hicks contractions, and pre-labor membrane rupture [2,39]. Research into the timing and frequency of membrane sweeping to enhance its effectiveness and acceptability in contemporary obstetrics practice is valuable. Based on the above observations, the present study aimed to evaluate the effectiveness of single fetal membrane sweeping at term in reducing the incidence of elective labor induction for postdate pregnancies and assess its impact on maternal and perinatal outcomes.

## Materials and methods

Literature review search strategies

The identification of articles was conducted through multiple formal search methods, including manual searches of key journals and textbooks, as well as electronic searches of databases using specific keywords, author names, and reference scanning. Electronic databases such as Google Scholar, PubMed, Wiley Online, Sci-Hub, Web of Science, and the Cochrane Database were systematically searched. Keywords employed in the search included "membrane sweeping," "post-term pregnancy," "postdate pregnancies," and "elective induction of labor." Initially, these searches produced over 250 articles across many databases, with some being unrelated to the study's focus. However, refining the search with phrases like "membrane sweeping," "prevention of post-date pregnancies," and "elective induction of labor at term" narrowed the results down to approximately 100 articles, predominantly consisting of older publications (published more than 15 years ago).

The criteria for selecting articles included prospective randomized controlled trials (RCTs) and case-control studies involving low-risk pregnant women at term, with gestational age determined through date calculation or early ultrasonography. The interventions compared membrane sweeping to control groups, with outcomes including the number of women initiating spontaneous labor or requiring formal induction of labor for postdate pregnancies. Cochrane reviews and meta-analyses of RCTs were also considered. Articles and textbooks had to be original, peer-reviewed, local, or international research studies presented at conferences or published as technical reports. Exclusion criteria involved studies with unknown sources or those unrelated to the subject matter, as well as older publications.

Effectiveness of membrane sweeping on prevention of postdate pregnancies and formal labor induction

Currently, the National Institute for Health and Care Excellence recommends membrane sweeping to stimulate spontaneous labor before resorting to formal labor induction, based on several RCTs and Cochrane reviews demonstrating its benefits [40]. Various studies have employed different methods to assess the effectiveness of membrane sweeping. Some have reported the number of women entering spontaneous active labor, while fewer have reported elective labor inductions. In these studies, membrane sweeping consistently appears beneficial in reducing the incidence of postdate pregnancies.

Effect of membrane sweeping on the onset of spontaneous labor at term

An Indian study by Saichandran et al. [41] found that serial membrane sweeping every 48 hours from 40 weeks until labor onset or up to 41 weeks gestation significantly reduced the incidence of postdate pregnancies and the need for labor induction. They reported that 98% of women who underwent membrane sweeping went into spontaneous labor, compared to 46% of women who did not. However, this reduction in elective labor induction was a presumption rather than a confirmed outcome, and maternal satisfaction was not assessed. A local study by Mehmood and Bashir [42] also observed that spontaneous labor occurred in 76% of women in the membrane-sweeping group compared to 38% in the control group, leading to a significant reduction in the need for labor induction (11% vs. 26%). Still, in Pakistan, a case-control study [43] reported that 77.83% of women presented in spontaneous labor following serial membrane sweeping from 40 weeks gestation, compared to 62.6% in the control group, but the proportion of women who eventually required elective labor induction was not stated. An older study by Woong et al. [44], however, suggested that a single episode of membrane sweeping beyond 40 weeks gestation did not reduce formal labor induction. Our study aimed to explore whether single membrane sweeping might be more effective at earlier gestational ages.

Effect of membrane sweeping on the incidence of elective labor induction and maternal satisfaction

Elective labor induction at term is a key strategy for preventing complications related to post-maturity. However, both clinicians and patients express concerns about the risks associated with labor induction, including an increased risk of cesarean section. To avoid formal labor induction and encourage spontaneous labor at term, membrane sweeping has been recommended [40]. Although results are inconsistent, some evidence suggests that serial membrane sweeping at term may reduce the need for labor induction in postdate pregnancies.

In an attempt to avoid one formal labor induction, Boulvain et al. [39] noted that serial membrane sweeping must be performed on eight women. A study in Nigeria by Iferikigwe et al. [45] found that only 11.3% of patients who underwent membrane sweeping subsequently required elective labor induction for postdate pregnancy, compared to 37% in the control group. More recent studies including one conducted in Abuja, Nigeria, have reported similar results [46-48]. However, the study by Iferikigwe et al. involved serial membrane sweeping, while the Abuja study employed a single fetal membrane sweep. Zamzami and Senani in Saudi Arabia [47] reported that 81.3% of women entered spontaneous labor following a single fetal membrane sweep, with only 8.75% requiring repeat membrane sweeping. However, the studies did not assess the rate of elective labor induction for those women who had a single episode of membrane sweep [45-56]. Our study aimed to explore whether single membrane sweeping may be more effective and acceptable to both patients and clinicians.

Influence of confounding variables on the outcome of membrane sweeping

It is well established that variables such as parity, gestational age, Bishop score, and gravidity can influence the onset of spontaneous labor and the length of term pregnancy. Although nulliparous women tend to have a higher risk of prolonged pregnancy than multiparous women, Yildirim et al. [52] found that membrane sweeping was equally effective for both nulliparous and multiparous women. In the study by Aliya and Rubina [55], no statistically significant difference was observed between nulliparous and multiparous patients. However, a study by Azra et al. [56] on multiparous women found no benefit of membrane sweeping compared to the control group. This might be attributed to the fact that most participants in the study already had a favorable Bishop score at recruitment, eliminating the need for membrane sweeping. The timing of recruitment and the initial Bishop score could significantly impact the results, with recruitment at 38 weeks possibly providing a more representative sample of women likely to experience postdate pregnancy and benefit from membrane sweeping.

Effect of timing of elective labor induction on fetal and maternal outcomes

The definition of postdate pregnancy varies across studies, with some considering pregnancies exceeding 40 or 41 weeks gestation, while others using a threshold of 42 weeks gestation based on departmental protocols. Studies have shown that fewer women required elective labor induction at 41 weeks when the induction was scheduled at 41+3 weeks gestation, leading to more favorable perinatal outcomes. This study will adopt induction of labor at 41+3 weeks for women who present at ≥41+3 weeks gestation and are not in labor, based on the departmental protocol at Central Hospital Benin City, Nigeria.

Safety of membrane sweeping

The safety of membrane sweeping is a critical consideration. Reported maternal risks include vaginal bleeding, irregular uterine contractions, and premature rupture of membranes, while fetal risks include meconium-stained amniotic fluid, neonatal infection, poor Apgar scores, and neonatal intensive care unit admission. Pre-labor membrane rupture is one of the complications that need careful attention, as it could offset the benefits of membrane sweeping. This study defined premature rupture of membranes as membrane rupture in the absence of uterine contractions and cervical dilatation for more than 12 hours.

Research gap

While several studies have studied the effectiveness of membrane sweeping, the results have been inconsistent across the globe. This has necessitated the need to evaluate the effectiveness of membrane sweeping in a Nigerian population. Thus, the primary objective of this study was to evaluate the effectiveness of single fetal membrane sweeping at term in reducing the incidence of elective labor induction for postdate pregnancies.

Justification

Managing postdate pregnancies can be challenging for obstetricians and healthcare systems. The standard approach in well-equipped centers involves selective induction of labor, which utilizes antepartum fetal testing to reduce the risk of complications from expectant management. However, this method is expensive and often unavailable in rural settings where many pregnant women are located. Routine induction, on the other hand, involves using mechanical or pharmacological agents to stimulate uterine contractions for vaginal delivery, but it is associated with an increased risk of adverse fetal and maternal outcomes. Therefore, there is a need for alternative methods to prevent postdate pregnancies and the complications associated with routine induction of labor.

One potential approach to preventing postdate pregnancies is to induce all patients before reaching 42 weeks of gestation. While this may seem reasonable, it comes with its own set of risks and costs, such as prolonged hospital admission and the need for induction of labor. A more desirable method is to use techniques that stimulate the spontaneous onset of labor, thus avoiding formal induction. Several minimally invasive techniques have been recommended, including membrane sweeping, unprotected coitus, and acupuncture. Membrane sweeping aims to prevent patients from going postdate and reduce the need for more formal methods of labor induction to minimize adverse effects and costs. In a country like Nigeria, where many people belong to the low socioeconomic class and cannot afford expensive methods of labor induction, membrane sweeping can emerge as a safe, cost-effective, and affordable alternative. Additionally, it has been suggested that repeated membrane sweeping may not provide significant benefits in terms of the need for postdate induction, while a single attempt at membrane sweeping may be more effective. Single membrane sweeping may also be more acceptable to patients, potentially leading to improved maternal satisfaction rates. However, many pregnant women are doubtful about the benefits of membrane sweeping, and their hesitancy increases when they are told that membrane sweeping will be repeated. Discomfort associated with the procedure further contributes to patient hesitancy. Therefore, conducting a study on a Nigerian population to assess the outcomes of single fetal membrane sweeping at term can provide valuable data to enhance patient counseling and decision-making. As such, the aim of this study was to evaluate the effectiveness of single fetal membrane sweeping at term in reducing the incidence of elective labor induction for postdate pregnancies to ultimately contribute to improved maternal and perinatal outcomes.

Research hypothesis

Research Question

Does single fetal membrane sweeping at term reduce formal labor induction for postdate pregnancies? 

Null Hypothesis 

There is no statistically significant increase in the number of spontaneous labors occurring before 41+3 weeks gestation in women who received a single fetal membrane sweep compared to those who did not receive a sweep between 38+0 to 40+6 weeks gestation. 

*Alternate Hypothesis* 

There is a statistically significant increase in the number of spontaneous labors occurring before 41+3 weeks gestation in women who received a single fetal membrane sweep compared to those who did not receive a sweep between 38+0 to 40+6 weeks gestation.

Aims and objectives

The primary aim of this study was to evaluate the effectiveness of single fetal membrane sweeping in reducing the incidence of elective labor induction for postdate pregnancies in a Nigerian population. Additionally, the secondary objective of this study was to evaluate the impact of single-fetal membrane sweeping on maternal and perinatal outcomes.

Specific objectives

The first specific objective of this study was to review existing literature on the effectiveness of fetal membrane sweeping in reducing the need for labor induction in postdate pregnancies, while the second specific objective was to determine the incidence of elective labor induction at 41+3 weeks gestation in women who received a single fetal membrane sweep between 38-40+6 weeks gestation and compare it to those who did not receive a sweep. Consequently, the third specific objective of this study was to document any adverse effects of single fetal membrane sweeping on both the mother and the baby. The final specific objective was to compare maternal satisfaction rates between women who received a fetal membrane sweep and those who did not. Based on the findings from objectives two, three, and four, the study will make appropriate recommendations.

Study background

This study was conducted at Central Hospital Benin City, a tertiary health institution in Edo State, Nigeria. The hospital serves as a vital center for providing specialized medical services to patients from Edo State and neighboring states, including Ondo, Delta, and Kogi. Central Hospital Benin City plays a crucial role in medical education and training for various healthcare professionals.

Pregnant women typically initiate antenatal care at Central Hospital Benin City around 20 weeks of gestation, with the majority of patients belonging to the low-income bracket, often engaged in trade. Antenatal booking registration is offered free of charge. The hospital records an average of 3,500 deliveries annually, with approximately 290 deliveries per month.

Notably, membrane sweeping is not routinely practiced at Central Hospital Benin City, as pregnancies are monitored until 41+3 weeks of gestation. If there are no signs of labor, elective induction of labor is performed. In cases of postdate pregnancies (≥ 41+3 weeks), women are commonly counseled to undergo intracervical extra-amniotic cervical ripening with a Foley catheter, aiming to improve the Bishop Score and make the cervix favorable for subsequent formal induction using artificial rupture of membranes (ARM) and synchronous oxytocin titration. In this regard, this study sought to explore an alternative approach that could reduce the need for formal induction of labor and its associated complications.

Study design and population

The study was designed as an open-label superiority RCT with no blinding. The study population included 140 low-risk pregnant women at term who met specific inclusion criteria.

Study inclusion and exclusion criteria

**Figure 1:**
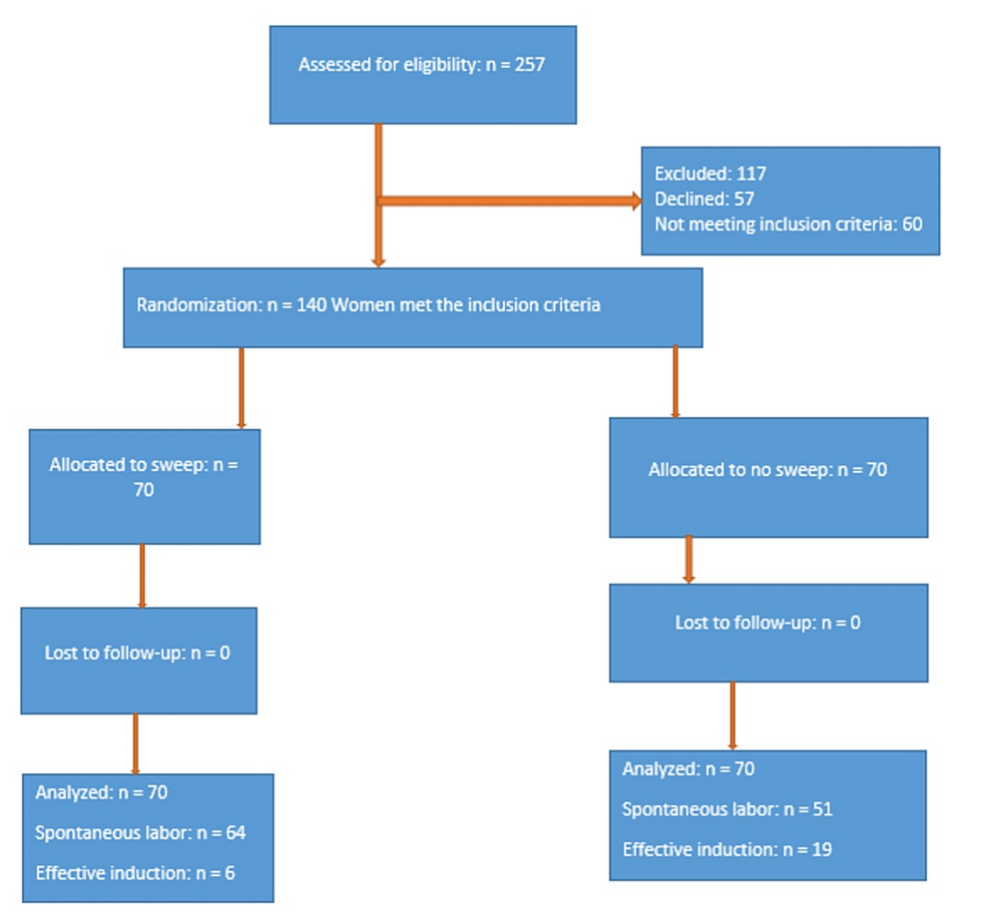
Study flow diagram indicating the study inclusion and exclusion criteria

For this study, the inclusion criteria included low-risk women with singleton pregnancies; cephalic presentation; longitudinal lie with intact fetal membranes; gestational age between 38+0 to 40+6 weeks, as determined by reliable last menstrual period (LMP) or early pregnancy ultrasound scan (done at not more than 16 weeks gestation); no previous cesarean delivery or uterine scar; Bishop score of <7; and no contraindication to vaginal delivery. Consequently, the exclusion criteria included women with a previous uterine surgery; twin gestation; intrauterine fetal demise (IUFD); premature rupture of membranes (PROM), undilated cervical os; abnormal fetal lie and presentation; estimated fetal macrosomia; gross fetal anomaly; medical disorders in pregnancy; contraindications to vaginal delivery; unreliable gestational age; and patients' refusal to participate in the study. The inclusion and exclusion criteria for this study have been aptly captured in Figure 1.

Sampling technique and recruitment

The recruitment of participants was carried out through routine antenatal health talks at Central Hospital Benin City. Women receiving antenatal care were educated about the study, and individual evaluations and counseling were conducted approximately one week before recruitment. This approach allowed participants enough time to make informed decisions. Further, the eligible participants were identified by the research team, which included the principal investigator and research assistants from the various units of the department. These research assistants were trained to assist with patient selection, recruitment, and data collection, all while adhering to the research objectives and protocol. Additionally, the participants were recruited at gestational ages of 38-40+6 weeks. Informed consent was obtained in writing during the antenatal visits at this gestational age.

Sample size

The sample size was calculated using the formula for a superiority randomized study for dichotomous variables as shown below: N = total sample size for both groups; n1 = sample size for group one; n2 = sample size for group two; Z1-α/2 = standard normal deviate at 95% confidence interval (1.96); Z1-β = standard normal deviate at 90% power of the study (0.90); p1 = proportion of the characteristics of interest in the target population for group one (12.4%); p2 = proportion of the characteristics of interest in the target population for group two (37.1%); P = total proportion of characteristics in both groups (12.35%); N = 4(1.96 +0.90)2(12.35)(100-12.35) (37.1 -12.35)2; N = 4(8.1796)(12.35)(87.65) 612.5625; N = 35416.932 / 612.5625; N = 57.8; N = 57.8 (with 20% attrition rate (69)).

Therefore, N = n1 + n2 = 138; n1 = n2 = 69; the number of participants for each group (group A, B) = 69 participants, approximating to 70 in each group, giving a total of 140 participants. The sample size was calculated using the formula for superiority randomized studies for dichotomous variables, taking into account a 20% attrition rate. The final sample size was determined to be 140 participants (70 in each group).

Study duration

The study was conducted over a ten-month period, from June 2020 to March 2021. Recruitment averaged 14 women per month or four women per week. It's important to note that the study duration was extended due to the impact of the COVID-19 pandemic. The study protocol involved a comprehensive clinical history of each participant, emphasizing factors such as age, gravidity, parity, last menstrual period (LMP), past obstetric and medical history, family and social history, and history of the current pregnancy. Routine antenatal blood investigations were reviewed, and gestational age was confirmed through ultrasound scans or accurate recall of the last menstrual period. Third-trimester ultrasound scans were performed or reviewed to assess fetal weight and placental localization. Further, the participants meeting the inclusion criteria were randomized into two groups, Sweep and No Sweep, using computer-generated random numbers, with a 1:1 ratio. The random numbers were placed in sealed envelopes that were sequentially numbered.

For the participants assigned to the Sweep group, membrane sweeping was performed by gently separating the forewater membrane from the lower uterine segment with two circumferential passes of the index fingers. The control group (No Sweep) underwent Bishop scoring only, and no further examinations were conducted until they entered spontaneous labor or reached 41+3 weeks gestation. Patients with closed cervical os were excluded. Additionally, following the procedure, a sanitary pad was placed on the vulva, and participants were advised to take paracetamol for any discomfort. The participants were counseled on what to expect, and who to contact following membrane sweeping. They were also placed on a fetal kick chart. All participants were instructed to present for elective induction of labor if they had not entered labor by 41+3 weeks gestation.

Labor and delivery

Participants presenting to the labor ward and delivery suite were assessed for estimated gestational age, reasons for admission (such as labor pains, induction, or liquor drainage), and Bishop scores. Those in the active phase of labor were admitted and monitored using a partograph. Moreover, data entry was conducted on a weekly basis and occasionally revised by the principal researcher. It is noteworthy that in this study, failure to achieve spontaneous labor by any arm of the study at 41+3 weeks gestation (recruitment to delivery interval of ≥ 10-24 days) was considered a postdate pregnancy and necessitated formal induction of labor using cervical ripening with a Foley catheter, followed by ARM and oxytocin titration. Further, continuous cardiotocography was employed to monitor fetal well-being, and cesarean sections were performed for cases of failed induction, prolonged labor, or fetal distress. Finally, following delivery, data were collected to assess labor outcomes, including the route and mode of delivery, maternal and neonatal complications (Apgar scores at first and fifth minutes, neonatal intensive care unit admissions), birth weight of babies, and maternal satisfaction. Consequently, maternal satisfaction was assessed using the PESPC (Patient Expectation and Satisfaction with Prenatal Care Instrument) questionnaire, consisting of four subscales: information, provider care, staff interest, and system characteristics.

Statistical analysis

The data collected was analyzed using IBM SPSS Statistics for Windows, Version 23, (Released 2015; IBM Corp., Armonk, New York, United States). Categorical variables were presented as numbers and percentages, while continuous variables were presented as means and standard deviations. Chi-square or Fisher's exact test was used to compare the proportions of categorical variables, and the Student's t-test or Mann-Whitney U-test was employed for continuous variables. Relative risks (RRs) with 95% confidence intervals were calculated, and a p-value less than 0.05 was considered significant.

Ethical consideration

Ethical approval for this study was obtained from the Research and Ethics Committee of the Hospital Management Board. The study adhered to ethical principles, including autonomy, beneficence, non-maleficence, and justice, in its interactions with human subjects. Informed consent was obtained from the participants, and strict adherence to the principles of minimal harm and patient well-being was upheld throughout the study. The study aimed to distribute benefits and burdens fairly among participants to ensure equal opportunities for all eligible individuals.

## Results

Characteristics of study participants according to group

The mean maternal age was 30.39±5.17 years in the membrane sweeping group and 30.34±5.56 years in the control group, with no significant difference observed (p=0.962). Additionally, both groups showed comparability in terms of parity, as indicated in Table 1. It's important to note that all p-values were > 0.05, signifying that the differences between both groups in baseline characteristics were not statistically significant. Regarding the mean gestational age at recruitment, it was 38.89±0.74 weeks for the membrane sweeping group and 38.92±0.80 weeks for the control group (p=0.85). In summary, as presented in Table 1, the baseline characteristics of the two groups were notably similar.

**Table 1:** Characteristics of study participants according to groups. p values > 0.05 are insignificant; p values derived with χ2 for categorical data and t-test for continuous variables. EFW: estimated fetal weight

Variable	Membrane sweeping (n=70)	Control (n=70)	p-value
Mean maternal age (years)	30.39±5.17	30.34±5.56	0.962
Gravidity	2.60±1.24	2.60±1.312	1.00
Parity			
Nulliparous	21 (30%)	18 (25.7%)	0.57
Primiparous	24 (34.3%)	29 (41.4%)	0.39
Multiparous	25 (35.7%)	23 (32.9%)	0.72
Mean gestational age at recruitment (weeks)	38.89±0.74	38.92±0.80	0.847
Cervical length (cm)	2.85±0.56	2.77±0.53	0.395
Pre-recruitment Bishop score	4.52±1.18	4.83±1.12	0.124
BMI (Mean+SD)	30.05±4.12	30.79±4.66	0.327
EFW (Mean+SD)	3.26 ± 0.41	3.68 ± 0.43	0.416

Recruitment to delivery time intervals

The results also revealed the distribution of subjects based on day intervals: one to seven, eight to 14, and ≥ 15. Interestingly, a higher number of women in the treatment group delivered within one to seven days of recruitment compared to their counterparts in the control group, and this difference was statistically significant (p=0.00001); however, the CI was not statistically significant (RR: 2.14 (95% CI: 0.49-3.05). However, the outcome numbers, i.e., 57 versus 20, may be clinically significant. 

Furthermore, there was a statistically significant difference between both groups regarding the recruitment to delivery interval of eight to 14 days, with a p-value of 0.0024 (RR: 0.33 (95% CI: 0.13-0.86)). 

The mean gestational age at delivery for women in the membrane sweeping group was 39.53±0.967 weeks, whereas for those in the control group, it was 40.63±0.88 weeks, resulting in a statistically significant difference (p=0.0001). This suggests that women in the control group were more likely to require elective labor induction for postdate pregnancy than those in the membrane-sweeping group.

In summary, among all participants who delivered at ≤41+3 weeks of gestation in both groups, membrane sweeping reduced the mean interval between intervention and delivery by 7.03 days (10.67±3.51 vs. 3.64±4.123; mean difference: 7.03, p=0.000). This underscores the effectiveness of membrane sweeping in expediting the delivery process (Table 2).

**Table 2:** Comparison of mean gestational ages at the delivery and the recruitment to delivery time intervals between the membrane-sweeping group and the control group. SD: standard deviation; RR: relative risk; CI: confidence interval

Outcome variable	Membrane sweeping group (n=70)	Control group (n=70)	RR (95% CI)	p-value
Mean gestational age at delivery (weeks)	39.53±0.967	40.63±0.88	-	0.000
Mean recruitment to delivery interval (days±SD)	3.64±4.123	10.67±3.51	-	0.000
1-7	57 (81.4%)	20 (28.6%)	2.14 (0.49-3.05)	0.00001
8-14	5 (7.1%)	15 (21.4%)	0.33 (0.13-0.86)	0.0024
≥15	2 (3.0%)	16 (22.3%)	0.09 (0.02-0.39)	0.0011

Obstetric outcomes: maternal outcomes, route of delivery, and mode of induction

Analyzing the data presented in Table 3, it becomes evident that the incidence of elective labor induction in the treatment group was notably lower, with six cases (9.0%), compared to the control group, which had 19 cases (27.1%). This significant difference in the incidence of elective labor induction yielded a p-value of 0.0083, along with an RR of 0.32 (0.13-0.74). Essentially, this implies that participants in the membrane sweep group had a 68% lower risk of elective labor induction than those in the control group. Furthermore, the results highlight that membrane sweeping significantly increased the likelihood of spontaneous labor. Women in the membrane sweeping group were significantly (p= 0.0054) more prone to experiencing spontaneous labor than those in the control group, with figures showing 64 women (91.4%) in the membrane sweep group versus 51 women (72.9%) in the control group, resulting in an RR of 1.25 (1.07-1.47).

**Table 3:** Obstetrics outcomes - maternal outcome, route of delivery, and mode of induction CPD: cephalopelvic disproportion; C/S: cesarean section; IOL: induction of labor; RR: relative risk; CI: confidence interval; ARM: artificial rupture of membranes

Outcome	Membrane sweeping	Control	RR (CI 95%)	p-value
Elective IOL (n, %)	6 (9.0%)	19 (27.1%)	0.32 (0.13-0.74	0.0083
Spontaneous labor	64 (91.4%)	51 (72.9%)	1.25 (1.07-1.47)	0.0054
Route of delivery	-	-	-	-
Vaginal	66 (94.29%)	61 (87.14%)	1.1 (0.97-1.20)	0.149
Spontaneous	64 (91.40%)	56 (80.00%)	1.14 (0.99-1.31)	0.0567
Assisted delivery (vacuum)	2 (2.90%)	5 (7.10%)	0.40 (0.08-1.99)	0.264
Indications for assisted delivery	-	-	-	-
Fetal distress	1 (1.42%)	3 (4.29%)	0.33 (0.04-3.17)	0.336
Maternal exhaustion	1 (1.42%)	1 (1.42%)	1.00 (0.06-15.67)	1.000
Prolonged second stage	0 (0.0%)	1 (1.42%)	0.33 (0.01-8.04)	0.499
C/S	4 (5.71%)	9 (12.9%)	0.44 (1.14-1.38)	0.159
Indications for C/S				-
Prolonged labor (CPD)	1 (1.42%)	4 (5.71%)	0.25 (0.03-2.18)	0.209
Fetal distress	1 (1.42%)	2 (2.86%)	0.50 (0.05-5.39)	0.568
Failed induction (cervical dystocia)	2 (2.86%)	3 (4.29%)	0.67 (0.11-3.87)	0.65
Postdate pregnancy	6 (9.00%)	19 (27.10%)	0.32 (0.13-0.74)	0.0083
Nulliparous	4/21 (19.04%)	10/18 (55.5%)	0.34 (0.13-0.91)	0.0312
Primiparous	2/24 (8.33%)	6/29 (35.7%)	0.40 (0.09-1.82)	0.237
Multiparous	0/25 (0%)	3/23 (13.4%)	1.13 (0.01-2.42)	0.17
Required induction	-	-	-	-
Yes	6	19	0.32 (0.13-0.74)	0.0083
No	0	0	-	-
Mode of labor induction at ≥41+3				
ARM+oxytocin only	4 (66.7%)	0 (0%)	25.7 (1.58-419.54)	0.0023
Foley catheter+ARM+oxytocin	2 (33.3%)	19 (100%)	0.33 (0.11-1.03)	0.0057

Looking specifically at delivery methods, 64 women (91.4%) in the membrane sweep group had spontaneous vaginal deliveries compared to 56 women (80.0%) in the control group. Although the p-value (p=0.0567) and the RR of 1.14 (0.99-1.31) is above the conventional threshold for statistical significance, the clinical outcome numbers support the observation that women in the study group are relatively more likely to have comparable spontaneous vaginal deliveries than women in the control group.

Assisted delivery through vacuum was recorded in two cases (2.90%) in the study group and five cases (7.10%) in the control group, resulting in an RR of 0.40 (0.08-1.99) and a p-value of 0.264. Major indications for assisted delivery included fetal distress in the second stage of labor (one case in the membrane-sweeping group and three cases in the control group), maternal exhaustion (one case in the membrane-sweep group and one case in the control group), and prolonged second stage (zero cases in the membrane sweeping group and one case in the control group).

Cesarean sections were performed on four women in the treatment group. Indications for these cesarean sections included prolonged labor due to cephalopelvic disproportion (one woman), fetal distress (one woman), and failed induction due to cervical dystocia (two women). In the control group, cesarean sections were conducted on four women who had prolonged labor due to cephalopelvic disproportion, two women with fetal distress, and three cases of failed induction due to cervical dystocia. Given that the differential risks for cesarean sections were not significantly different across the groups, it's noteworthy that the treatment and control groups showed comparable outcomes.

Moreover, when stratified according to parity, the positive effect of sweeping on the spontaneous onset of labor was particularly pronounced in nulliparous women. This was further supported by the fact that only four out of 21 nulliparous women (19.04%) in the treatment group compared to 10 out of 18 nulliparous women (55.5%) in the control group required elective labor induction for postdate pregnancies.

Finally, it's worth highlighting that women in the membrane sweep group were considerably less likely to undergo cervical ripening with a Foley catheter before labor induction than women in the control group, with figures indicating two cases (33.3%) versus 19 cases (100%). Though the difference was statistically insignificant, as indicated by the 95% CI in the RR of 0.33 (0.11-1.03), the p-value was significant, <0.05, and may have clinical significance when compared with the outcome numbers six vs. 19.

Maternal complications following membrane sweeping

Table 4 outlines the maternal complications associated with both the sweeping and control groups. Notably, there were no instances of clinical chorioamnionitis recorded in either of the two groups. Generally, when considering the occurrence of PROM before the onset of labor, there were no significant differences observed between the groups. Specifically, seven women (10%) in the treatment group experienced PROM, while 12 women (17.1%) in the control group faced a similar situation. The RR for this outcome was 0.58 (95% CI: 0.24-1.39), with a p-value of 0.225. Furthermore, when examining cases of ruptured membranes lasting for more than 24 hours, no instances were reported in either group. Additionally, there were no occurrences of accidental membrane rupture during or immediately after the membrane sweep procedure, and intrapartum pyrexia showed no significant differences between the groups.

**Table 4:** Maternal complications following membrane sweeping PPH: postpartum hemorrhage; RR: relative risk; CI: confidence interval

Maternal complications	Membrane sweeping (n=70)	Control (n=70)	RR (CI 95%)	p-value
Clinical chorioamnionitis	0 (0%)	0 (0%)	-	-
Premature rupture of membranes	7 (10.0%)	12 (17.1%)	0.58 (0.24-1.39)	0.225
Fever during labor				
Temperature (≤38^o^C)	1 (1.40%)	1 (1.40%)	1.00 (0.06-15.7)	1.000
Temperature (>38^o^C)	1 (1.40%)	0 (0%)	3.00 (0.12-72.4)	0.499
Meconium-stained amniotic fluid	0 (0.00%)	3 (4.30%)	0.14 (0.01-2.71)	0.195
Vaginal bleeding	7 (10.0%)	2 (2.86%)	3.50 (0.75-1.26)	0.110
PPH maternal satisfaction				
Completely dissatisfied	0 (0.00%)	1 (1.40%)	-	1.000
Dissatisfied	0 (0.00%)	2 (2.90%)	-	0.496
Neutral satisfied	3 (4.30%)	9 (12.90%)	-	0.128
Completely satisfied	21 (30.00%)	47 (67.10%)	-	0.000
Satisfied	46 (65.70%)	11 (15.70%)	-	0.000

On the whole, maternal complications were comparable between the two groups. It is worth noting that although minimal vaginal bleeding was reported more frequently by women in the stripping group (10% vs. 2.86%), this difference did not reach statistical significance, with a p-value of 0.110. Importantly, this bleeding was not severe enough to disrupt normal daily activities. Furthermore, there were no cases of meconium aspiration syndrome recorded, although three cases of meconium-stained amniotic fluid were observed in the control group. This difference was not statistically significant, with a p-value of 0.195. Additionally, women in the membrane sweep group expressed higher levels of complete satisfaction with the process, and this difference achieved statistical significance at <0.05. Importantly, there were no instances of post-partum hemorrhage observed in the study.

Neonatal outcomes and complications

The neonatal outcomes, which encompassed parameters such as the 1st and 5th-minute APGAR scores, birth weight, and the necessity for admission into the special care baby unit (SCBU), displayed slight variations between the two groups, although these differences did not attain statistical significance. Still, the incidence of macrosomia was identical in both groups. Specifically, the mean birth weight was 3.29±0.45 kg in the membrane sweep group and 3.31±0.42 kg in the control group (p=0.7861). Notably, one neonate in the sweep group and seven neonates in the control group had Apgar scores of less than 7 at five minutes (p=0.065). Furthermore, there were two (2.90%) admissions to the SCBU in the treatment group, in contrast to 10 (14.3%) SCBU admissions in the control group (p=0.0332). Regrettably, there was one case of perinatal death in the control group, while none were recorded in the treatment group. The data has been aptly captured in Table 5, which indicates the neonatal outcomes and complications.

**Table 5:** Neonatal outcomes and complications RR: relative risk; CI: confidence interval; SCBU: special care baby unit; MSAF: meconium-stained amniotic fluid; APGAR: appearance, pulse, grimace, activity, and respiration

Neonatal variable	Membrane sweeping	Control	RR (95% CI)	p-value
Birth weight (kg)	-			
<2.5	1 (1.40%)	1 (1.40%)	2.0 (0.19-2.90)	1.000
2.5-<4.0	65 (92.9%)	65 (92.9%)	1.0 (0.86-1.25)	0.688
Macrosomia ≥ 4	4 (5.70%)	4 (5.70%)	0.3 (0.04-3.03)	0.301
Mean birth weight	3.29 ± 0.45	3.31 ± 0.42	-	0.7861
One-minute APGAR score	-	-	-	-
<7	2 (2.90%)	8 (11.40%)	0.25 (0.06-1.14)	0.073
Five-minute APGAR score	-	-	-	-
<7	1 (1.40%)	7 (10.00%)	0.14 (0.02-1.13)	0.065
Neonatal pyrexia	2 (2.90%)	3 (4.30%)	0.67 (0.12-3.87)	0.651
MSAF	0 (0.00%)	3 (4.30%)	0.14 (0.01- 2.72	0.195
SCBU admission	2 (2.90%)	10 (14.3%)	0.20 (0.04-0.88)	0.0332
Perinatal death	0 (0.00%)	1 (1.40%)	0.33 (0.01-8.05)	0.499

Outcomes between parities in the membrane-sweeping group

As depicted in Table 6, the mean gestational age stood at 39.74±1.00 for nulliparous women and 39.45±0.95 for parous women. Notably, there was a slightly higher proportion of post-date pregnancies in the nulliparous group (four, 19.5%) compared to the parous group (two, 4.08%), although this disparity did not achieve statistical significance (p=0.062). Furthermore, an insignificant difference in the number of women experiencing spontaneous deliveries was observed across the groups: 81% in the nulliparous group in contrast to 96% in the parous group (p=0.123). However, although the nulliparous group had more assisted deliveries than the parous group, there was still no statistically significant difference between the two groups (5% vs. 2%, RR: 2.33 (95% CI: 0.15-35.6), p=0.542)).

**Table 6:** Outcomes according to parity *Mean GA at delivery PROM: premature rupture of membrane, GA: gestational age, SCBU: specialized care baby unit; RR: relative risk; CI: confidence interval

Variables	Nulliparous (n=21)	Parous (n=49)	RR (95% CI)	p-value
*GA at delivery	39.74 ± 1.00	39.45 ± 0.95	-	0.255
Elective labor induction	4 (19.05%)	2 (4.08%)	4.67 (0.92-23.5)	0.062
Route of delivery	-	-	-	-
Spontaneous delivery	17(80.95%)	47 (95.91%)	0.84 (0.68-1.05)	0.123
Assisted delivery	1(4.76%)	1 (2.04%)	2.33 (0.15-35.6)	0.542
Caesarian section	3 (14.29%)	1 (2.04%)	7.0 (0.77-63.5)	0.083
Maternal complications	-	-	-	-
PROM	1 (4.76%)	6 (12.24%)	0.37 (0.05-2.90)	0.945
Meconium-stained amniotic fluid	0 (0%)	0 (0%)	-	-
Vaginal bleeding	1 (4.76%)	6 (12.24%)	0.37 (0.05-2.90)	0.945
Maternal fever	0 (0%)	2 (4.26%)	0.45 (0.02-9.08)	0.605
Neonatal complications	-	-	-	-
Macrosomia	0 (0%)	4 (8.16%)	0.26 (0.01-4.49)	0.349
Neonatal pyrexia	1 (4.76%)	1 (2.04%)	2.33 (0.15-35.7)	0.542
SBCU admission	1 (4.76%)	1 (2.04%)	2.33 (0.15-35.7)	0.542

Regarding the instances of cesarean sections in the two groups, it was noted that three (14.29%) of the nulliparous women underwent the procedure compared to one (2.04%) parous woman; the RR was 7.0 (0.77-63.5), with p=0.083. In addition, the number of women experiencing PROM was higher in the parous group (12.24%) in comparison to the nulliparous women (4.76%), with an RR of 0.37 (95% CI: 0.05-2.90) and p=0.945. Moreover, there were four cases of fetal macrosomia among the parous women and none in the nulliparous group, while two parous women developed intrapartum fever, with none among the nulliparous group. Notably, there were no discernible differences in the number of neonates with neonatal pyrexia and admissions to the SCBU across different parity groups. This has been aptly captured in Table 6, which indicates the outcomes according to parity.

## Discussion

The primary focus of this study was to assess the impact of a single membrane sweeping procedure on the prevention of elective labor induction for postdate pregnancies. The results of this study demonstrated that the baseline characteristics of the two groups were similar, with no statistically significant differences. Key demographic factors, including maternal age, parity, gestational age, and Bishop Score at the study's commencement, were comparable between the groups. Additionally, factors such as marital status, level of education, and body mass index did not show any significant differences. It is also important to note that in this particular study, Bishop scores for the control group were determined before the inclusion of participants. This approach differed from some other studies that opted not to assess Bishop scores beforehand due to concerns that the procedure might stimulate the release of prostaglandins, potentially influencing cervical ripening and uterine contractions [41-57]. In this study, assessing the Bishop score was deemed necessary to control for and mitigate any potential bias or influence it might have on the study's outcomes. It was also essential to evaluate the Bishop score for the control group to ensure that the women met the inclusion criteria. Moreover, the randomization process during participant selection was implemented to minimize the impact of such a test procedure on the overall results, as observed in studies conducted by Ugwu et al., de Miranda et al., and Kashanian et al., where Bishop scores were assessed for both groups [45,51,57].

The study's findings revealed that single membrane sweeping significantly reduced the incidence of elective labor induction. Specifically, only 9% of women in the treatment group required elective labor induction compared to 27.1% in the control group. This suggests that women undergoing a single membrane sweeping procedure are significantly less likely to undergo elective labor induction. Based on the results and subsequent analysis, the null hypothesis was rejected, and the alternate hypothesis was accepted. These findings align with the results from previous studies. For instance, Iferikigwe's study conducted in Enugu State, Nigeria, [45] demonstrated that 11.3% of patients who had their membranes swept subsequently required elective labor induction for postdate pregnancy, compared to 37.7% of patients whose membranes were not swept. Similarly, a study by Nyamzi and colleagues in Abuja, Nigeria [46], reported significantly lower rates of elective labor induction in the membrane sweep group (12.4%) compared to the control group (37.1%). However, it's worth noting that Iferikigwe and co-workers employed repeated membrane sweeping, unlike the single membrane sweep approach utilized in this study and the Abuja study. Additionally, in the Abuja study, the membrane sweep groups exhibited a more favorable initial Bishop score than the control group, potentially introducing bias. In contrast, in this new study, there were no statistically significant differences in the recruitment Bishop scores between the treatment group and the control group (p=0.124).

Furthermore, it was observed that performing fetal membrane sweeping for five women (number needed to treat (NNT)) was necessary to prevent one formal labor induction. This finding contrasts with the Cochrane review conducted by Boulvain et al. [39], where the NNT was reported as eight women. The variation in these numbers may be attributed to the heterogeneity of the studies included in the Cochrane review. Also, the study found a statistically significant difference in the incidence of spontaneous labor between the treatment group (91.4%) and the control group (72.9%), with a p-value of less than 0.005. This outcome is consistent with the results of a study by Nasim et al., which reported that 56% of women went into spontaneous labor following a single episode of membrane sweeping [49]. The lower percentage in this study may be attributed to late recruitment, resulting in participants having a shorter period before induction of labor was considered. Additionally, Piriya's study in Thailand [50] reported a higher proportion of women delivering at ≤40 weeks gestation following a single membrane sweep at 38 weeks compared to no intervention (69.33% vs. 51.1%). These findings collectively underscore the effectiveness of membrane sweeping in promoting spontaneous labor onset, although the specific outcomes may vary based on the timing of recruitment and other contextual factors.

The higher rate of spontaneous labor observed in our study compared to previous research may be attributed to our departmental protocol, which schedules labor induction at 41+3 weeks of gestation, in contrast to the previous study where induction occurred at 40 weeks of gestation. Nyamzi and colleagues in Nigeria [46] noted that inducing labor at 41+3 weeks was well-received by both patients and clinicians. In their study, they found that following membrane sweeping, 87.6% of women went into spontaneous labor compared to 62.9% in the control group. Similar to our study, all women who were not in labor at 41+3 weeks of gestation were scheduled for induction. These findings also align with the research conducted by de Miranda et al. [57], which demonstrated that membrane sweeping significantly increased the incidence of spontaneous labor onset before 42 weeks of gestation, leading to a reduced need for formal labor induction at 42 weeks. It's worth noting that in de Miranda et al.'s study, serial membrane sweeping was performed, unlike our new study. Consequently, if a single episode of fetal membrane sweeping can produce a similar effect on the duration of term pregnancy as serial membrane sweeping, it would be a preferred choice for both patients and clinicians, potentially explaining the high rate of maternal satisfaction observed in the membrane sweep group in our study. In contrast, the study by Maryam Kashanian and colleagues in Iran [51] did not find any significant benefit of membrane sweeping in promoting spontaneous labor onset. This discrepancy may be attributed to the fact that most of their participants were primigravida with closed cervices who received cervical massage instead of membrane sweeping. Unlike our new study, these specific participant categories were excluded from our research, potentially contributing to the different outcomes observed.

A substantial difference was observed in the mean time interval from recruitment to delivery, referred to as the recruitment-delivery interval, between the control group (10.67±3.51 days) and the group that underwent membrane sweeping (3.64±4.123 days), with a p-value of < 0.005. Additionally, a significant reduction in the mean gestational age at delivery was noted (39.53±0.967 weeks vs. 40.63±0.88 weeks) when comparing the control group to the membrane sweeping group, with a p-value of 0.0000. These findings strongly suggest that women in the control group were considerably more likely to necessitate elective labor induction for postdate pregnancy in contrast to those in the membrane-sweeping group. Furthermore, the study revealed that membrane sweeping led to a remarkable reduction in the mean interval between randomization and delivery by seven days, a figure surpassing the results of similar studies conducted by Ugwu et al. [45] (reduction of three days), de Miranda et al. [57] (reduction of one day), Yildirim et al. [52] (reduction of four days), and Aliyu Yabagi Isah and colleagues in the federal capital territory, Abuja, Nigeria, where the recruitment-delivery interval was 3.35±2.55 days for those who underwent membrane sweeping and 5.76±2.75 days for those who did not. This variance in results could be attributed to the delayed recruitment of participants in our study.

It's noteworthy to mention that McColgin et al.'s study [33] also reported a seven-day difference, even though the treatment group had a significantly higher parity than the control group. Similarly, consistent seven-day earlier delivery in the membrane sweeping groups have been reported in different studies [58-60]. However, these findings contrast with Kashanian et al.'s study [51], where no statistically significant reduction in the time interval between sweeping (stripping) and vaginal examination till delivery was observed between the sweeping and control groups (7.7±6.9 days vs. 7.1±5.6 days, p=0.61), with a mean difference of 0.6, implying no significant statistical reduction in the time interval between recruitment and delivery in the two groups [51]. Putnam and colleagues [58] and Woong and coworkers [44] reported similar findings, with Woong specifically evaluating membrane sweeping beyond 40 weeks gestation and scheduling labor induction at 42 weeks gestation. However, this approach may not align with contemporary obstetrics practices. This study also observed that 81.4% of participants in the treatment group went into spontaneous labor within seven days of recruitment, in contrast to only 28% of women in the control group. A similar observation was made by Yildirim [52]. These consistent findings further underscore the importance of this procedure. Based on the aforementioned findings, it is evident that membrane sweeping leads to the spontaneous onset of labor and vaginal delivery within seven days. Therefore, it can be confidently asserted that a single fetal membrane sweeping at term constitutes an effective technique for preventing postdate pregnancies. This is particularly relevant in resource-poor settings in Sub-Saharan Africa where access to maternal and neonatal health services can be prohibitively expensive. This straightforward procedure can be performed by midwives or community health extension workers when properly trained, offering a viable alternative for women who prefer to avoid formal induction of labor [58-63].

In the discourse surrounding the effectiveness of membrane sweeping in preventing postdate pregnancies, considering the results of this study alongside similar research in the literature, it becomes apparent that the findings on the efficacy of membrane sweeping have been inconsistent and sometimes contradictory. Some studies have suggested that membrane sweeping can reduce the incidence of postdate pregnancy [25,30,33], while others, such as Kashanian, Tan, and Hamdan et al., have reported that sweeping does not significantly reduce the time to delivery [51,63-68]. Furthermore, a recent Cochrane review and meta-analysis of 14 RCTs concluded that membrane sweeping at 38 weeks gestation onwards did not appear to yield clinically significant benefits. This conclusion contradicts the results of a more recent RCT involving women at 41 weeks gestation, which found that the procedure substantially reduced the incidence of postdate pregnancy [32].

In attempting to elucidate the conflicting reports in the literature, it's worth noting that de Miranda et al. pointed out that the Cochrane review encompassed studies characterized by relatively small sample sizes and significant heterogeneity among the included studies [57]. Furthermore, an earlier study conducted by Woong et al. [44] demonstrated that a single episode of fetal membrane sweeping beyond 40 weeks of gestation did not lead to a reduction in formal induction of labor. Given these observations and the fact that our study specifically evaluated the efficacy of single fetal membrane sweeping in a subgroup of women whose pregnancies had progressed to 38 weeks and beyond but had not yet reached the post-date stage, it is conceivable that this subgroup of term pregnant women may require more time for membrane sweeping to affect the Bishop score compared to women who are already post-date [52]. This distinction could contribute to the significantly favorable outcomes we observed in our study. It is also crucial to emphasize, as previously argued by Nyamzi et al. [46], that when analyzing the results of studies involving single or serial membrane sweepings from 41 weeks onwards, the procedure is typically performed as an outpatient intervention without the immediate goal of labor induction. This practice is justified by the fact that perinatal morbidity and mortality begin to increase even before 41 weeks of gestation [46]. Consequently, results derived from studies following this methodology, which deviates from those where sweeping was initiated from 38 weeks onwards, may account for discrepancies in outcomes compared to our study.

In our investigation, women in the study group were significantly more likely to experience vaginal deliveries than those in the control group. The data strongly indicated that membrane sweeping substantially enhanced the likelihood of spontaneous vaginal delivery. These findings align with a study conducted by Sachandrian et al. [41], where a majority of patients in the study group also achieved vaginal deliveries [41]. Further, in their study, a total of 50 women underwent membrane sweeping in the study group, while another 50 women in the control group did not undergo this procedure. The results of their study, which are in line with the findings of our present study, demonstrated that within the study group, 47 women (94%) experienced spontaneous labor and subsequently had vaginal deliveries. In contrast, among the control group, only 23 women (46%) initiated spontaneous labor, out of which 22 eventually had spontaneous vaginal deliveries.

Our study's findings also revealed that postdate women in the membrane sweeping group were less likely to require cervical ripening with a Foley catheter compared to women in the control group. This significant reduction in the need for cervical ripening with a Foley catheter, as demonstrated in our study, implies that the risks and costs associated with using this intervention can be substantially mitigated through membrane sweeping. However, it's worth noting that these results contrast with those of Anna Lemonte and Joseph Miller [6], whose study suggested that membrane stripping had only a limited effect on reducing the need for cervical ripening with a Foley catheter. Still, in our study, although there was no significant difference in the risk of cesarean section between the two groups, the treatment group did exhibit a reduction in the incidence of cesarean sections. This aligns with the findings of Nyamzi et al. [46], who also observed a clinically significant decrease in the rate of cesarean deliveries with membrane stripping. However, due to limitations in sample size, Nyamzi et al. suggested that larger trials were needed to confirm this finding. Similarly, Ugwu et al. [45] found that membrane stripping did not appear to increase the rate of cesarean sections, as the route of delivery was comparable to the control group. In contrast, Tarik and Zamzami [47] reported an increased risk of cesarean delivery in the stripped group, although this difference did not reach statistical significance. Further, the impact of membrane stripping on the rate of cesarean deliveries remains inconclusive at this point and warrants further investigation through larger-scale trials. Nevertheless, efforts to reduce the cesarean section rate and associated risks at Central Hospital Benin are strongly encouraged and advocated.

In this study, as previously reported in similar research [44-46], no cases of clinical chorioamnionitis were observed in either of the two groups. This finding contrasts with the results of Kathleen Putnam et al [58], who reported an elevated risk of clinical chorioamnionitis in the membrane-stripped group (10.3% vs. 6.0%). Hend and colleagues [61] also noted two pregnancies in the stripped group complicated by intrapartum chorioamnionitis, compared to only one in the control group, though the results were not statistically significant (p>0.05). These discrepancies may be attributed to the multiple membrane sweep procedures conducted in their respective studies. Further, the results of our study indicated no significant differences across the groups in terms of obstetric outcomes, such as PROM before the onset of labor. In the stripped group, seven women experienced PROM compared to 12 in the control group, with an RR of 0.575 (95% CI: 0.241-1.374) and p=0.323. This outcome aligns with the findings of Saichandran et al. [41], where two patients had PROM followed by spontaneous onset of labor and normal vaginal delivery. Yildirim and Turham [52] reported a slightly higher occurrence of PROM in the stripped group (13.4% vs. 9.6%), although the difference was not statistically significant (p=0.266). Nyamzi et al [48] found equal rates of PROM in both groups (8.2% vs. 8.2%). Notably, authors reporting a significant increase in PROM following membrane sweeping typically performed serial sweeps, whereas our study involved a single sweep. The primary rationale for performing fetal membrane sweeping is to reduce the incidence of postdate pregnancies and subsequent elective labor induction. However, if this objective results in a higher rate of labor induction for PROM, it would undermine the perceived benefits of the procedure.

The occurrence of vaginal bleeding was reported more frequently by women assigned to membrane sweeping (10% vs. 2.9%) but did not reach statistical significance (p=0.1654). This finding is consistent with the results reported by de Miranda et al [57], where the bleeding was more common in the sweeping group (although the p-value was not provided in their study). Similar results were also observed in Ugwu et al. [45], where two women in the membrane-swept group experienced mild vaginal bleeding compared to none in the control group. It should be noted that the study was not adequately powered to evaluate the association between membrane sweeping and vaginal bleeding, as acknowledged by the authors. Yildirim and colleagues [52] also noted that 26.8% of women in the stripped group reported slight vaginal bleeding (p=0.225). In summary, while there is a slight increase in the likelihood of vaginal bleeding and discomfort associated with membrane sweeping, these effects are not statistically significant enough to be considered problematic. Most women are willing to endure this mild discomfort in anticipation of the procedure's benefits, as noted by Hend S Saleh et al. [61], who stated that many patients and physicians would consider a small risk worthwhile when a considerable advantage was anticipated. Hence, membrane sweeping can be considered a safe clinical procedure. Our study also found that participants expressed greater satisfaction with the birth process following a single membrane sweep compared to the control group, although maternal discomfort was not specifically assessed. This finding aligns with the observations made by Nyamzi and colleagues, who noted that women in the sweeping group expressed greater satisfaction despite experiencing slightly more initial discomfort after membrane sweeping.

To determine which factors influenced the likelihood of labor induction for women who remained undelivered at 41+3 weeks, we conducted univariate and multivariate Cox regression analyses of simultaneous factors. The results revealed that spontaneous onset of labor before 41+3 weeks was significantly associated with gravidity, gestational age at recruitment, and membrane sweeping (p<0.05). Similar findings were reported by Yildirim and colleagues. Putnam et al. identified the pre-treatment Bishop Score as the sole significant factor in their study, although the notable difference in the initial Bishop score between the groups served as the primary limitation in their research. However, in our study, we observed no cases of meconium-stained amniotic fluid in the study group, contrasting with a rate of 4.3% (three cases) in the control group. This difference was not statistically significant (p=0.2446). This finding contradicts the results reported by Sachandrian [41], where five patients in the treatment group had meconium-stained amniotic fluid compared to 27 in the control group. Kashanian et al. [51] also reported meconium-stained amniotic fluid in 13 women, with eight in the sweeping group and five in the control group, showing no statistically significant differences between the two groups (p=0.39).

Regarding neonatal outcomes, no significant differences in fetal outcomes and complications were observed between the groups. Consequently, we can conclude that there is no evidence to suggest that the adjunct outpatient procedure of membrane sweeping increases the risk of maternal or fetal infection, a conclusion that aligns with findings from several other studies [38,39,41,45,46]. Thus, the practice can be affirmed as safe and effective in preventing elective labor induction. Moreover, theoretically, it has been argued that membrane sweeping should be more beneficial in parous women [32]. However, in our study, the positive effects of membrane sweeping were observed regardless of parity. Concerning the effect of membrane sweeping, we noted that there were more women with postdate pregnancies requiring labor induction in the nulliparous group (four out of 21, 19.05%) than in the parous group (two out of 49, 4.08%). Nevertheless, this difference was not statistically significant (p=0.062). Additionally, a significant difference was found in the number of women with spontaneous delivery across the groups, with a higher percentage of women in the parous group (95.91%) experiencing spontaneous vaginal deliveries. These findings corroborate those of a study by de Miranda et al. [57], where RR reduction was more substantial in parous women, which indicates a significant positive effect of membrane sweeping on spontaneous onset of labor.

Our study did observe a discrepancy in the rates of cesarean sections between the two groups, with three out of 21 nulliparous women (14.29%) undergoing a cesarean section compared to one out of 49 parous women (2.04%). These findings are consistent with the results of a study by Levine et al. [65], which investigated the risk of cesarean delivery among both nulliparous and multiparous women. Based on their results, it was concluded that irrespective of parity, the risks were similar. Regarding other risks and complications, conditions such as vaginal bleeding, PROM, macrosomia, neonatal pyrexia, and SCBU admission were more prevalent among the parous group compared to the nulliparous group. It is important to note that when discussing the effect of membrane sweeping in relation to parity, these outcomes pertain to subgroup analysis, and the statistical power to detect small differences is limited. Nevertheless, among the notable clinical significance of the present study is the observation that single fatal membrane sweeping is not only safe but also an effective method of inducing labor in post-date pregnancies. Single fetal membrane sweeping has been noted to have a higher rate of success with regard to the attainment of vaginal delivery, even as it has a lower rate of both fetal and maternal complications, which makes it an important and increasingly attractive alternative to clinical practice, especially in low-resource and low-income settings. The safety and efficacy of single-fetal membrane sweeping have further been aptly supported by the absence of fetal and maternal deaths. Single fetal membrane sweeping may, therefore, be used in reducing formal induction of labor in post-date pregnancies.

Strengths and limitations of the study

This study derives its strength from its design as an RCT. Notably, the baseline characteristics of the participants in both groups were similar, reducing the potential for bias. To maintain objectivity, the Bishop score was assessed for both groups, and women with a closed cervix in either of the groups were excluded from the study. Maternal satisfaction was evaluated by trained midwives who were unaware of the randomization process, ensuring impartiality in the assessment. Additionally, the allocation concealment was overseen by an independent statistician, further enhancing the study's credibility.

Consequently, a number of limitations were noted in the present study, including the use of a minimum sample size, which may not fully represent the broader population of pregnant women. Employing a larger sample size could enhance the external validity of the findings and expand the scope of conclusions that can be drawn. The subgroup analysis was another notable limitation, given that, in discussing the effect of membrane sweeping concerning parity, it's essential to acknowledge that these outcomes are related to subgroup analysis. It's worth noting that the statistical power to detect subtle differences in this context is relatively low. The third notable limitation regards the clinical protocol. Thus, the departmental protocol at Central Hospital Benin City stipulates that pregnancies at or beyond 41+3 weeks should undergo induction of labor. This protocol aims to prevent women from reaching 42 weeks of gestation and mitigate the potential risks associated with prolonged pregnancy. However, this aspect of the protocol imposes limitations on the study. Cervical parameters may improve over time, and if women were followed up until 42 weeks, more of them might go into spontaneous labor, potentially yielding more favorable results than those reported in this study. Still, the study design was another notable limitation. For instance, in this study, a single sweeping strategy was compared to no sweeping at all. Consequently, the study design does not allow for conclusions about whether single fetal membrane sweeping is superior to the serial sweeping protocols utilized in other studies. Lastly, the lack of blinding was another major limitation in the present study. It is worth noting that this study was not blinded. The lead investigator was directly involved in conducting the procedure, collecting data, and was aware of the study objectives. This lack of blinding could introduce some degree of bias into the study's outcomes.

Recommendations

The results of this study provide strong evidence that membrane sweeping is both safe and effective in reducing the need for elective labor induction in postdate pregnancies. Therefore, its use in obstetrics as a means to prevent elective labor induction is not only justified but also warrants careful consideration, particularly as a cost-effective alternative, especially in healthcare settings with limited resources. Midwives, in particular, should be encouraged to incorporate this procedure into their practice. Additionally, the findings from this study open up several promising avenues for further research. It would be valuable to explore the effects of cervical massage and other cervical ripening methods, such as the gradual insertion of the examining finger into the cervix until the membranes can be swept. Comparing these techniques and their outcomes in terms of their ability to separate the membranes from the lower uterine segment during the initial examination could provide valuable insights into their roles in expediting the onset of labor.

Furthermore, future studies should assess the impact of membrane sweeping on maternal discomfort and pain. Understanding the experiences of women undergoing this procedure can lead to improvements in patient care and comfort during pregnancy and labor. Another area worth investigating is the potential synergistic effects of membrane sweeping when combined with other induction methods. Exploring how membrane sweeping interacts with other techniques and its impact on the production of substances like relaxin, phospholipase A2, and oxytocin could provide insights into its ability to ripen an unfavorable cervix and potentially reduce the need for supplemental oxytocin during labor. Given the significant results realized in relation to the secondary outcomes of this study, it is recommended that prospective studies should be developed to assess the above findings as primary research outcomes. Such studies should not only have larger sample sizes within specific healthcare environments but also be conducted in different locations to enhance the external validity of the findings. This will help validate or challenge the conclusions drawn from this study, further contributing to the body of knowledge in this field and enhancing the quality of obstetric care.

## Conclusions

The findings of this study revealed that the baseline characteristics of the groups were comparable, with statistically insignificant differences between them. Notably, membrane sweeping emerged as a significant factor in reducing the incidence of elective labor induction. As a result, the null hypothesis of the study was rejected. Furthermore, the results demonstrated that membrane sweeping significantly heightened the likelihood of spontaneous labor onset, subsequently leading to a reduction in the mean gestational age at delivery among women in the membrane-sweeping group. Additionally, the study found that postdate women in the membrane sweep group were less likely to require cervical ripening with a Foley catheter before labor induction involving amniotomy and synchronized oxytocin titration, compared to women in the control group. Regarding maternal complications, the results of this study indicated that there were no significant differences between the groups. Although minimal vaginal bleeding was reported more frequently by women subjected to membrane sweeping, these effects did not reach statistical significance. Thus, they were not deemed problematic. In terms of neonatal outcomes, it can be inferred that no significant differences were observed in fetal outcomes and complications across the groups. Remarkably, the positive impact of membrane sweeping was evident regardless of parity, even though more women in the parous group experienced spontaneous vaginal deliveries compared to nulliparous women. 

In summary, the study results confirmed that membrane sweeping yielded clinically significant benefits by reducing the incidence of postdate pregnancies and enhancing the occurrence of spontaneous vaginal deliveries. Importantly, there was no evidence to suggest that this ancillary outpatient procedure increased the risk of maternal or fetal complications. This conclusion aligns with findings from several other studies, underscoring the practice's safety and its significant role in preventing elective labor induction for postdate pregnancies. Nevertheless, the present study has presented a number of strengths and limitations. Among the notable strengths include the observation that the use of RCT as the study design for the examination of the intervention is appropriate and ensures that the study is of high quality. Also, the use of RCT aids in ensuring that the groups under study are comparable in relation to the known and unknown baseline factors. Further, the study has utilized a design that entails the randomization of the study groups into treatment and comparator groups with concealed allocations, which aids in minimizing selection biases. However, the present study also has a number of limitations, including the observation that it has been conducted in one geographical location (Nigeria), which has limited the representation of the sample and affects the generalizability of the study findings. The other limitation includes the observation that the study heavily depended on the self-report of the patients for certain variables, which might introduce certain biases in the data.
